# 1574. Patient, Health System, and Clinical Encounter Characteristics Associated with Use Of Antibiotics Without A Prescription In The United States

**DOI:** 10.1093/ofid/ofac492.103

**Published:** 2022-12-15

**Authors:** Larissa Grigoryan, Michael K Paasche-Orlow, Osvaldo Alquicira, Kenneth Barning, Hamad Mahmood, Thomas Porter, Fareed Khan, Juanita Salinas, Jean Raphael, Michele Fowler, Richard Street, Barbara Trautner

**Affiliations:** Baylor College of Medicine, Houston, Texas; School of Medicine, Boston University, Boston, Massachusetts; Baylor College of Medicine, Houston, Texas; Baylor College of Medicine, Houston, Texas; Baylor College of Medicine, Houston, Texas; Baylor College of Medicine, Houston, Texas; Baylor College of Medicine, Houston, Texas; Baylor College of Medicine, Houston, Texas; Baylor College of Medicine, Houston, Texas; Memorial Hermann Health System, Katy, Texas; Michael E. DeBakey Veteran Affairs Medical Center, Center for Innovations in Quality, Effectiveness, and Safety (IQuESt), Houston, Texas; Michael E. DeBakey Veterans Affairs Medical Center / Baylor College of Medicine, Houston, TX

## Abstract

**Background:**

Use of antibiotics without a prescription (purchased in the United States (U.S.), purchased in other countries, or obtained from friends and relatives) is potentially unsafe and may increase the global risk of antimicrobial resistance. We evaluated the effect of patient, health system, and clinical encounter factors on intention to use antibiotics without a prescription.

**Methods:**

Waiting room survey conducted from January 2020 and March 2021 in three continuity and three same-day public primary care clinics and two private emergency departments. Non-prescription use was defined as the consumption of antibiotics not prescribed for the individual’s current condition. Intended use was defined as professed intention to take antibiotics if feeling sick, without contacting a doctor, nurse, or clinic, in any of the following situations: 1) buying antibiotics without a prescription in the U.S., 2) buying antibiotics without a prescription in another country, and 3) getting antibiotics from friends or relatives. The effect of patient, health care system, and clinical encounter factors were studied using multivariate logistic regression.

**Results:**

Of the 564 respondents, 247 (43.8%) reported prior use of non-prescription antibiotics; half of these instances involved penicillins (mostly amoxicillin). Overall, 177 (31.4%) of the respondents intended to use antibiotics without a prescription from one of the three sources (Figure 1). Younger age, lack of health insurance and high cost of doctor visits were predictors of intention to buy antibiotics without a prescription in the U.S. **(**Table 1**)**. Predictors of intention to buy antibiotics without a prescription from other countries included younger age, being interviewed in Spanish, and reporting that a language barrier is a major problem for medical appointments. Lack of health insurance and high cost of physician visits were associated with the intention to use antibiotics obtained from relatives or friends.

**Conclusion:**

Interventions aimed at reducing non-prescription antibiotic use should focus on addressing language barriers, ensuring health insurance coverage, and reducing financial barriers to primary care visits.

**Disclosures:**

**Larissa Grigoryan, MD, PhD**, Rebiotix Inc: Grant/Research Support **Michael K. Paasche-Orlow, MD, MPH**, GlaxoSmithKline: Advisor/Consultant **Barbara Trautner, MD, PhD**, Genetech: Advisor/Consultant.

